# Miscellaneous

**Published:** 1891-09

**Authors:** 


### MISCELLANEOUS.

### A CURIO'S LETTER.

Among the papers in the possession of the New Haven Colony Historical Society, is a letter from Benjamin Franklin to Ralph I. Ingersoll, dated Philadelphia, December 11, 1762. The following is an extract from the letter: I should be glad to know what it is that distinguishes Connecticut religion from common religion:—communicate, if you please, some of these particulars that you think will amuse me as a virtuoso. When I traveled I thought of your excessively strict observation of Sunday; and that a man could hardly travel on that day among you upon his lawful occasions without Hazard of Punishment, while where I was every one traveled, if he pleased, or diverted himself in any other way; and in the afternoon both high and low went to the Play or the Opera, where there was plenty of Singing, Fiddling and Dancing. I looked around for God's Judgments, but saw no signs of them. The Cities were well built and full of Inhabitants, the Markets filled with Plenty, the people well favored and well clothed; the Fields well tilled; the Cattle fat and strong; the Fences, Houses and Windows all in repair; and no Old Terror any where in the country,—which would almost make one suspect that the Deity is not so angry at the offense as a New England Justice.

### CELERY.

Probably no class of people suffer more with rheumatism than farmers, and yet the remedy for this dreadful disease is, or should be, right at hand. If celery were eaten freely, sufferers from rheumatism would be comparatively few. It is a mistaken idea that cold and damp produce the disease; they simply develop it. Acid blood is the primary and sustaining cause. If celery is eaten largely, an alkaline blood is the result, and where this exists there can be neither rheumatism nor gout. It should be eaten cooked. Cut it into bits, and boil till soft, in as little water as possible. Add to this half as much milk as there is water in the celery, thicken with flour, and season with butter, pepper, and salt. If you cook it nicely and give it a fair trial, I am sure you will as soon leave potatoes out of the daily bill of fare as celery. It is nice as sauce for any kind of cold meat or fowl, or for roasted poultry or game of any kind. Children will like it poured over boiled potatoes, or it may be drained from the sauce, mixed with mashed potatoes, formed into little cakes and browned. A ready witted woman will find numerous ways of using it.—*Melbourne Weekly Times.*

### SPEED OF THE ELECTRIC CURRENT.

Professor Marks, of the Edison Electric Light Company, is authority for the assertion that if the globe was encircled with a continuous cable a current would travel the entire distance in a trifle over three seconds. At this rate a current would travel to the sun, covering the entire distance of 96,000,000 miles, in three and a half minutes.

We are yet in our infancy, as far as electricity goes. New discoveries will yet be made, and we will live to see them put into practical use, which will revolutionize the entire world. The experiment which we are about to make in telegraphy is only a feeler which will lead to other and more startling experiments. The establishment of telephone communications between the hemispheres is already being seriously discussed.

### TO TAN SKINS WITH FUR, HAIR OR WOOL ON.

If the skin is dry, first soak it in soft water until pliable. Take a straight piece of an old scythe, one and a half or two feet long. Dull about four or six inches of each end, insert them in wooden handles, or wind them with cloth, so as not to injure the hands. Lay the skin upon a bench or inclined board with the flesh side up, and remove all flesh and fat with the above instrument; also trim off the skirts or rough edges. Mix together pulverized alum one pound, salt one-half pound, wheat bran one pint. Add sufficient water to make a thick paste, which spread over the flesh side of the skin as evenly as possible; fold it, bringing the flesh side in, roll up closely, and lay away where it cannot freeze for about four days; then unroll, brush off the paste, work the skin with the hands until pliable.

### THE FIRST MEERSCHAUM PIPE.

In 1723 there lived in Pesth, the capitol of Hungary, Karol Kowates, a shoemaker, whose ingenuity in cutting and carving on wood, etc., brought him in contact with Count Andrassy, ancestor to the Prime Minister of Austria, with whom he became a favorite. The Count, on his return from a mission to Turkey, brought with him a large piece of whitish clay, which had been presented to him as a curiosity, on account of its extraordinary light specific gravity. It struck the shoemaker that, being porous, it must naturally be well adapted for pipes, as it would absorb the nicotine. The experiment was tried, and Karol cut a pipe for the Count, and one for himself. But in the pursuit of his trade he could not keep his hands clean, and many a piece of shoemaker's wax became attached to the pipe. The clay, however, instead of assuming a dirty appearance, as was naturally to be expected, when Karol wiped it off, received, wherever the wax had touched, a clear brown polish, instead of the dull white it previously had. Attributing this change in the tint to the proper source, he waxed the whole surface, and, polishing the pipe, again smoked it, and noticed how admirably and beautifully it colored ; also, how much more sweet the pipe smoked after being waxed. Karol had struck the smoking philosopher's stone : and other noblemen, hearing of the wonderful properties of this singular species of clay, imported it in considerable quantities for the manufacture of pipes. The natural scarcity of this much esteemed article, and the great cost of importation, in those days of limited facilities for transportation, rendered its use exclusively confined to the richest European noblemen until 1830, when it became a more general article of trade. The first meerschaum pipe made by Karol Kowates has been preserved in the museum of Pesth.

One of the great American beauties of olden times was Sally Ward, of Louisville. She married several times, and at 50 was as charming as ever. Josephine, at 45, was the loveliest in a court of beauty. To-day the Princess of Wales is one of the most beautiful women in England, yet she is a grandmother, and is 45, if she is a day.

The Brain.—It is not intellectual work that injures the brain, says the London *Hospital*, but emotional excitement. Most men can stand the severest thought and study of which their brains are capable, and be none the worse for it; for neither thought nor study interferes with the recuperative influence of sleep. It is ambition, anxiety, and disappointment, the hopes and fears, the loves and hates of our lives, that wear out our nervous system and endanger the balance of the brain.

Lupus.—Pyrogallic acid has astringent properties, and is an excellent dressing for ulcers of any nature not specific in character. Salicylic acid, in powdered form, answers better for specific ulcers than any local application. Dust this on in the evening, permit it to remain all night, and wash it off in the morning, then reapply again at night. Few specific ulcers resist its action.

Washing White Sheep Skin Mats.—Boil some soap in a little water so as to make a strong lather; mix this up in a sufficient quantity of water, rather more than lukewarm, to wash the mat in, more boiled soap being rubbed on those portions of it which may require additional cleansing. When the mat has been well washed in this water, another must be prepared in the same way, and a second washing takes place, followed by a third, which should be sufficient to clean it thoroughly. It must be then rinsed in cold water until all the soap is removed, and put into another water with just enough blue in it to keep the wool a good white, and prevent its inclining to yellow. After this it should be thoroughly wrung, shaken and hung out in the open air to dry, with the skin part towards the sun, but not where it is scorching, otherwise it will become hard. It must be frequently shaken while drying (if not it will be quite crackly) and must also be frequently turned—that is, hung up first by one end and then by the other, until perfectly dry.

To Wash Delicate Colored Muslins.—Boil wheat bran (about two quarts to a dress) in soft water half an hour; let it cool, strain the liquor and use it instead of soap suds; it removes dirt like soap, keeps the color and the clothes only need rinsing in one water, and starching is unnecessary. Suds and rinsing water for colored articles should be used as cold as possible. Another way is to make thick corn meal mush, well salted, and use instead of soap; rinse in one or two waters, and do not starch.

Mud Cure for Snake Poison.—A colored boy near Newberne, N. C., was bitten recently by a rattlesnake, and, as medical aid was out of reach, the boy must have surely died but for the thoughtfulness of his companions. They dug a hole in the ground and placed both legs in it up to near the hips, and packed the mud securely around him. The poison was entirely extracted, and the boy is now well.

Root Beer.—Take one ounce each of sassafras, allspice, yellow dock and wintergreen, one-half ounce each of wild cherry bark and coriander, one-fourth ounce hops and three quarts molasses. Pour sufficient boiling water on the ingredients, and let them stand twenty-four hours, filter the liquor and add one-half pint yeast, and it is ready for use in twenty-four hours.

To Case-Harden Articles.—To case-harden articles with prussiate of potash, they should be first polished and afterward heated to a bright red, when they should be rubbed over with a mixture of three parts of prussiate of potash and one of sal ammoniac. When cooled to a dull red, immerse in water.

To Prevent Frosting on Glass.—A very thin coating of glycerine applied to glass will prevent frost forming on it in the coldest weather. This is specially interesting to engineers, who are much annoyed in frosty or foggy weather by the forming of a film on their instruments.

To Take Out Paint.—Equal parts of ammonia and turpentine will take paint out of clothing, no matter how hard or dry it may be. Saturate the spot two or three times, then wash out in soap-suds.

Papering Old Wall.—For a whitewashed wall to be papered, sponge first with good vinegar. This kills the lime, and the paper will not peel off.

To Harden Wood.—Wood steeped in a solution of iron sulphate or copperas becomes very hard, and almost indestructible.

